# Associations between pre-cue parietal alpha oscillations and event related desynchronization in motor imagery-based brain-computer interface

**DOI:** 10.3389/fnhum.2025.1625127

**Published:** 2025-07-23

**Authors:** Mohamed A. Mohamed, Joshua Giles, Mashael AlSaleh, Mahnaz Arvaneh

**Affiliations:** ^1^School of Electrical and Electronics Engineering and Neuroscience Institute, University of Sheffield, Sheffield, United Kingdom; ^2^Department of Information Technology, College of Computer and Information Sciences, King Saud University, Riyadh, Saudi Arabia

**Keywords:** event-related desynchronization, MI, brain-computer interface, pre-cue, parietal alpha

## Abstract

**Introduction:**

Motor Imagery based brain-computer interfaces (MI-BCIs) offer a promising avenue for controlling external devices via neural signals generated through imagined movements. Despite their potential, the performance of MI-BCIs remains highly variable across users and sessions, presenting a barrier to broader adoption.

**Methods:**

This study explores the influence of pre-cue parietal alpha power on the quality of the event-related desynchronization (ERD) responses, a critical indicator of MI processes. Analyzing data from 102 sessions involving 77 participants.

**Results:**

We identified a robust significant correlation between pre-cue parietal alpha power and ERD magnitude, indicating that elevated pre-cue parietal alpha power is associated with enhanced ERD responses. Additionally, we observed a significant positive relationship between pre-cue parietal alpha power and MI-BCI classification accuracy, highlighting the potential relevance of this neurophysiological metric for BCI performance.

**Discussion:**

Our findings suggest that pre-cue parietal alpha power can serve as a potential marker for optimizing MI-BCI systems. Integrating this marker into individualized training protocols can potentially enhance MI-BCI systems' consistency, and overall accuracy.

## 1 Introduction

A brain-computer interface (BCI) is a system that enables users to control external devices using their brain signals, which can be recorded non-invasively using methods like electroencephalogram (EEG) (Singh et al., 2021). In a MI -based BCI (MI-BCI), the user imagines performing different body movements in order to control an external device (Arvaneh et al., 2017). Similar to actual movements, MI modulates sensorimotor rhythms (SMR), consisting of mu (8–13 Hz) and beta (13–30 Hz). During MI, there are changes in these rhythms that are known as event-related desynchronization (ERD) and event-related synchronization (ERS) (Rimbert et al., 2022; Pfurtscheller et al., 2006).

ERD presents a reduction in the SMR amplitudes in the contralateral motor cortex (Pfurtscheller et al., 1996). In the context of MI, ERD is often associated with the preparation and mental simulation of motor movements (Pfurtscheller et al., 1996). ERS, on the other hand, is seen as an increase in the SMR power. ERS often occurs in the same hemisphere as the imagined movement and is typically observed after an ERD (Pfurtscheller et al., 1996; Neuper et al., 2006; Thomas et al., 2013; Pfurtscheller et al., 2006; Gwon and Ahn, 2021).

MI-BCIs typically rely on detecting ERD patterns, the primary neural signature of MI, as the control signal (Thomas et al., 2013). Pfurtscheller and Da Silva (1999) introduced a method to quantify ERD as the percentage change in SMR power during MI compared to the baseline, where the baseline is defined as the time interval prior to the MI instruction signal (Tangwiriyasakul et al., 2013). Previous studies showed that stronger ERD can reflect focused mental effort and better control, while weaker ERD can indicate less engagement or mental fatigue (Pfurtscheller et al., 1996).

Although MI-BCIs hold great promise, their practical usability remains limited due to significant challenges users face in achieving and sustaining effective performance (Khosla et al., 2020). Between 10% to 30% of users are often unable to gain effective control of the BCI, struggling to reach the threshold performance level of over 70% (Thompson, 2019; Zhang et al., 2020).

Existing literature suggests that the limited reliability of MI-BCI control can be attributed to two primary factors(Jeunet et al., 2017): (1) algorithm inaccuracy, stemming from limitations in machine learning and signal processing algorithms, and (2) user training inefficiency. Although substantial efforts have been dedicated within the BCI community to enhance BCI algorithms, the equally important factor of user training has often received comparatively less attention (Roc and Lotte, 2020). Long-term BCI training has been shown to induce measurable neural adaptations, highlighting the importance of sustained practice for skill acquisition (Tortora et al., 2022). The primary goal of user training is to enhance BCI control abilities by helping users develop effective mental strategies (Roc et al., 2021). Without the ability to consistently produce distinguishable and stable EEG patterns during different mental tasks, even the most advanced machine learning algorithms will struggle to accurately detect and interpret user intentions (Roc et al., 2021).

However, suboptimal user training, especially when not tailored to individual abilities, can lead to substantial variability in MI-BCI performance (Rimbert et al., 2022). The current training process is often time-consuming and resource-intensive (Ahn and Jun, 2015). Leveraging predictions of control ability based on pre-cue brain states, the neural activity observed before MI initiation, offers the potential to improving BCI training and may provide insight into why some users struggle with standard protocols (see Section 4 for further discussion). This approach can inform the selection of suitable BCI paradigms and the adaptation of training protocols to better align with individual needs. Adjustments could include optimizing interface design, refining instructions, modifying event timing, and customizing feedback types (Bamdadian et al., 2014).

Previous studies have identified two main categories of BCI performance predictors: neurophysiological and psychological (Ahn and Jun, 2015). Among psychological factors, Nijboer et al. (2010) found that motivation and confidence positively correlated with BCI performance, while fear exhibited a negative correlation. However, these psychological predictors were primarily assessed through self-reported measures and involved a limited number of participants, raising concerns about their objectivity and potential inaccuracies (Bamdadian et al., 2014).

In addition to the psychological predictors, various neurophysiological predictors have been investigated by BCI researchers. Blankertz et al. (2010) proposed a neurophysiological predictor based on mu and beta rhythms extracted via EEG from the sensorimotor cortex during a two-minute eyes-open relaxed state. Their investigation observed a positive significant correlation between their proposed predictor and MI-BCI classification accuracy (Blankertz et al., 2010). Maeder et al. (2012) validated neurophysiological predictors similar to those proposed by Blankertz et al. (2010), at both the participant and trial levels (Maeder et al., 2012). Notably, the predictors in this study were extracted during the pre-cue phase rather than during an eyes-open resting state. They found that BCI performance predictability was higher at the trial level than at the participant level, likely because trial-level predictors were derived immediately before task initiation during the pre-cue interval, offering a closer temporal link to subsequent performance.

Likewise, Ahn et al. demonstrated that users with poor BCI performance exhibited significantly lower alpha and higher theta power across multiple states (non-task-related state, resting state before task onset, and during the MI state). Theta differences were most pronounced in the frontal and central areas, while alpha differences were significant across much of the scalp. Based on their findings, a predictor of BCI classification accuracy using the relative power levels of alpha, beta, theta, and gamma bands was proposed. Their predictor slightly outperformed the previously proposed model (r = 0.59). Similarly, Grosse et al. proposed neurophysiological predictors calculated during the pre-cue phase, focusing on the relationship between gamma oscillations and SMR power. Their study observed a positive correlation between frontal and occipital gamma oscillations and SMR power while noting a negative correlation between central parietal gamma oscillations and SMR power (Grosse-Wentrup and Schölkopf, 2012).

In contrast, Bamdadian et al. conducted a trial-level analysis, investigating the relationship between BCI classification accuracy and a novel pre-cue predictor, i.e. the ratio of frontal theta power to the sum of central beta and parietal alpha powers. Their findings showed a positive correlation between this predictor and BCI performance (Bamdadian et al., 2014). More recently, Marissens Cueva et al. introduced an approach using ERD induced by brief post-median nerve stimulation as a predictor of MI-BCI expertise. Their results showed that post-median nerve stimulation-induced ERD could classify users into low and high performer groups with up to 74% accuracy, offering a potential neurophysiological marker for early user stratification (Cueva et al., 2025).

Despite these efforts, some of the aforementioned neurophysiological predictors have often yielded inconsistent results across studies and have struggled to replicate reliably (Jeunet et al., 2015). This inconsistency may be due in part to the use of classification accuracy as the primary performance metric—a measure influenced by factors such as the number of training trials, task complexity, and feature extraction methods or averaging steps (e.g., averaging ERD or spectral power values across sessions) (Lotte and Jeunet, 2017; Rimbert et al., 2022). As a result, understanding the neurophysiological underpinnings of BCI control remains a complex challenge, underscoring the need for alternative approaches that account for temporal dynamics and individual variability.

Rather than refining existing participant-level or resting-state predictors, the present study adopts a trial-by-trial perspective, focusing on neural activity immediately preceding MI. Specifically, we investigate fluctuations in parietal alpha power during the pre-cue interval—a temporally aligned and cognitively meaningful marker that may reflect a user's attentional readiness and mental state prior to task execution (Sharma and Singh, 2015). Distinct from earlier approaches that primarily focused on predicting classification accuracy, our study examines the direct relationship between pre-cue neural activity and the strength of ERD, the core signal used in MI-BCI control.

Focusing on pre-cue parietal alpha power offers new opportunities for developing real-time, user-adaptive BCI systems. MI trials could be dynamically initiated when users are in a cognitively receptive state, thereby reducing low-quality attempts and enhancing training efficiency. Feedback strategies and training protocols could also be tailored based on momentary mental readiness, such as signs of fatigue or disengagement. Substantial evidence links parietal alpha activity to attentional focus, cognitive workload, and readiness to perform tasks (Sauseng et al., 200; Klimesch, 2000; Sharma and Singh, 2015); however, these findings have largely remained theoretical or limited to offline analyses. There is a growing need to translate this knowledge into practical BCI applications that proactively respond to users' cognitive states prior to task execution. This shift toward cognitively aware, flexible BCIs emphasizes not only performance outcomes but also the brain's preparatory state.

Within this context, our study investigates the relationship between parietal alpha power during the pre-cue interval and the strength of ERD in the motor cortex during MI. Consistent and robust ERD features are essential for effective MI-BCI performance (Rimbert et al., 2022; Ahn and Jun, 2015), and are known to be influenced by attentional and cognitive states prior to task onset (Bamdadian et al., 2014; Wang et al., 2023). Parietal alpha power, in particular, has been identified as a neural correlate of these states, including sustained attention, mental effort, and relaxation (Sauseng et al., 200; Klimesch, 2000; Sharma and Singh, 2015), all of which support optimal ERD generation.

Although our investigation is not yet integrated into real-time BCI systems, it represents a foundational step toward operationalizing pre-cue parietal alpha power as a reliable neural marker for BCI readiness. By analyzing this relationship at the trial level, we aim to inform the design of adaptive BCI protocols that can respond proactively to users' mental states.

To test this hypothesis, we analyzed three MI datasets comprising 102 sessions from 77 participants. One of these datasets, which was previously analyzed in Bamdadian et al. (2014)'s study. By evaluating how variations in pre-cue parietal alpha power relate to the strength of ERD during MI, this study contributes to a deeper understanding of ERD variability and supports ongoing efforts to improve the consistency and reliability of MI-BCI performance.

## 2 Methodology

### 2.1 Datasets

This research combined three datasets that adhered to a similar MI-BCI protocol, selected based on specific criteria to ensure the comparability of EEG data after processing. The key selection criteria were as follows: (1) Among the many MI-EEG datasets available online, only those using the standard Graz protocol were included. This protocol is widely adopted in MI-BCI research and features predefined MI tasks, such as hand or foot movements, designed to elicit distinct ERD/ERS pattern. (2) Each MI task needed to last at least 3 seconds to provide enough data samples for reliable ERD analysis. (3) All participants were required to be healthy to minimize potential confounding effects from neurological or neuromuscular disorders on brain signals. (4) The datasets had to include recordings from EEG channels Pz, C3, and C4. The Pz channel was used to calculate pre-cue parietal alpha power, whereas C3 and C4, key electrodes positioned over the brain's sensorimotor area, were essential for analyzing ERD changes during MI tasks.

A total of 102 sessions that met the criteria were identified, with around 70% coming from publicly available data to ensure the study's replicability. These sessions were denoted as A_*i, j*_ for dataset 1, B_*i, j*_ for dataset 2, and C_*i, j*_ for dataset 3 where *i* and *j* represent the participant and session numbers, respectively. A detailed description of these datasets is provided in Section 2.1.1.

#### 2.1.1 Description of datasets

**Dataset A (Cho et al., 2017)**: In this dataset, EEG data were collected from 52 participants (19 females and 33 males, average age ± SD = 24.8 ± 3.86 years). Among the participants, two (A_20_ and A_30_) were left-handed, while the remaining 50 were right-handed. EEG recordings were obtained using 64 Ag/AgCl active electrodes arranged according to the international 10-10 system, with a sampling rate of 512 Hz. Before the MI experiment, participants practiced moving their fingers, starting from the index finger to the little finger and touching each to their thumb within three seconds after onset. Following this practice, participants were instructed to imagine hand movements as per the given instructions. Each MI experiment consisted of five or six runs. During these runs, participants completed either 100 or 120 trials of left-hand MI and 100 or 120 trials of right-hand MI. All EEG trials were high-pass filtered above 0.5 Hz to remove drifts.

**Dataset B (Bamdadian et al.**, **2014****)**: In this dataset, 16 healthy participants, including two left-handed individuals, were recruited. EEG data were recorded using 27 channels arranged according to the international 10-10 system across two separate sessions conducted on different days, sampled at a rate of 250 Hz. The first session served as a calibration session, while the second was a non-feedback session. Participants were explicitly instructed to minimize physical movements and eye blinking during the EEG recording to reduce potential artifacts. During the experiment, a visual cue on the computer screen directed participants to either engage in MI or remain idle. In the MI trials, participants were instructed to imagine kinaesthetic hand movements corresponding to their handedness (left or right). During idle trials, participants performed mental counting to ensure consistency and minimize variability. Each MI trial lasted 10 seconds, and the experimental design included two runs per session. Each run consisted of 80 trials, equally divided between MI and idle conditions.

**Dataset C**: The last dataset utilized in this paper was sourced from the BCI Competition IV (Brunner et al., 2008). This dataset includes EEG recordings from nine healthy participants, captured using 22 Ag/AgCl electrodes positioned according to the international 10–20 system and sampled at a rate of 250 Hz. During the MI sessions, participants performed four specific MI tasks: imagining movements of the right hand, left hand, feet, and tongue. These MI sessions were held on different days, with each participant undergoing two sessions, each consisting of six runs with short breaks in between. Each run included 12 trials for each MI task, totaling 288 trials per session. The EEG signals were recorded monopolarly, with the left mastoid as the reference and the right mastoid as the ground. The signals were bandpass-filtered between 0.5 and 100 Hz, and a 50 Hz notch filter was applied to eliminate line noise.

To reduce differences between the datasets, this study concentrated exclusively on MI-EEG datasets obtained through the established MI-BCI paradigm detailed by Pfurscheller and Neuper (Neuper et al., 2006). Referred to as the Graz protocol, this paradigm entails a binary MI task where participants are directed to imagine movements.

This study included only MI tasks related to imagining left and right-hand movements. Each trial began with a fixation cross displayed on the screen for 2 s, accompanied by a beep sound to prepare the participant for the upcoming task. Following the fixation cross, a cue was presented, indicating which hand (left or right) the participant should imagine moving. This cue was displayed for 1 second. After the cue, the participant engaged in the MI task for 3 seconds (from 1 to 4 s). During this period, the participant focused on mentally simulating the movement of the indicated hand. Following the MI task, the participant was allowed to relax for approximately 6 seconds, allowing the participant to rest before the next trial began.

### 2.2 EEG processing

**Figure 1:**
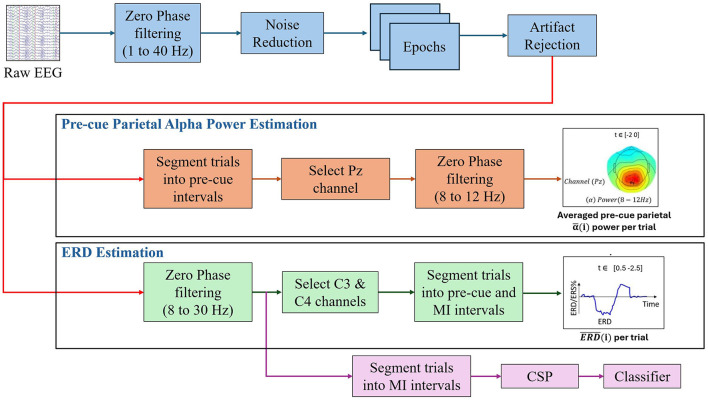
Overview of the EEG data processing and analysis pipeline used in this study.

All data analysis, including signal preprocessing, artifact rejection, epoch segmentation, statistical analysis, and topographical scalp plots, was performed in MATLAB 2023b and EEGLAB toolbox. A detailed pipeline was established to standardize the data processing workflow (see Figure 1). The process included all steps necessary to prepare and analyze EEG data.

The pipeline began with bandpass filtering the raw EEG signals using zero-phase elliptic bandpass filters within a frequency range of 1 to 40 Hz. After filtering, the common average reference (CAR) was applied due to its proven effectiveness and low computational complexity, as outlined by Tsuchimoto et al. (2021). The continuous EEG data from each session were then segmented into epochs spanning from -2.5 s to 3.5 s relative to the cue onset. This time window was specifically selected to encompass both the baseline and MI periods, aligning with the experimental design described in detail in later sections. Next, artifact rejection was performed by identifying and excluding trials in which the signal amplitude exceeded ±120 μV at any electrode, as recommended by Wang et al. (2023).

#### 2.2.1 Calculation of pre-cue parietal alpha power

Our study aimed to examine how variations in pre-cue parietal alpha (α) power influence the quality of ERD responses observed during MI tasks. Specifically, we calculated the average alpha power during the pre-cue interval at channel Pz for each trial *l*, denoted as ${\bar{\alpha }}_{i,j}^{l}$, where *i* and *j* represent the session and participant identifiers, respectively.

As described in the preprocessing step, the baseline pre-cue interval was established as −2 to 0 s relative to cue onset. To compute ${\bar{\alpha }}_{i,j}^{l}$, a zero-phase elliptic bandpass filter (8–12 Hz) was first applied to the interval −2.5 to 0.5 s relative to the cue onset for each trial *l* at channel Pz. To avoid potential distortions caused by the filtering process, the first and last 0.5 s of the filtered interval were excluded from further analysis. This step ensured that any edge effects introduced by the filter do not affect the subsequent estimation of alpha power. Finally, the average power of the filtered signal within the baseline interval −2 to 0 s (relative to the cue onset) was calculated and recorded as ${\bar{\alpha }}_{i,j}^{l}$.

#### 2.2.2 ERD estimation

To estimate ERD, we first applied a zero-phase elliptic bandpass filter from 8 to 30 Hz to the extracted time interval, as described in the preprocessing section. To reduce signal distortion effects due to filtering, we excluded the initial and final 0.5 s of the epochs. Thereafter, the baseline interval was selected from −2 to 0 s (referred to as **BL**), and the MI interval from 0.5 to 2.5 s (referred to as **X**), where 0 marks the cue onset for imagined movement.

For each trial *l*, time instance *s*, and channel *ch*, the ERD percentage was computed as:


(1) $$\begin{array}{l} {\text{E}\text{R}\text{D}}_{i,j}^{l}\left(s,ch\right)=\frac{{\left[{\text{X}}_{i,j}^{l}\left(s,ch\right)\right]}^{2}-\bar{{\left[{\text{B}\text{L}}_{i,j}^{l}\left(ch\right)\right]}^{2}}}{\bar{{\left[{\text{B}\text{L}}_{i,j}^{l}\left(ch\right)\right]}^{2}}}\times 100, \end{array}$$

Where ${\text{X}}_{i,j}^{l}\left(s,ch\right)$ represents the bandpass-filtered EEG amplitude at time sample *s* and channel *ch* during the MI interval, with *ch* corresponding to C3 for right-hand and C4 for left-hand. The term $[{\text{X}}_{i,j}^{l}\left(s,ch\right){]}^{2}$ corresponds to the instantaneous power of the bandpass-filtered EEG signal during the MI interval. Finally, $\bar{[{\text{B}\text{L}}_{i,j}^{l}\left(ch\right){]}^{2}}$ denotes the average power of the bandpass-filtered EEG signal during the baseline interval.

Equation 1 defines the percentage change in sensorimotor oscillatory power, where negative values represent ERD and positive values represent ERS. As highlighted in prior research (Pfurtscheller and Da Silva, 1999; Rimbert et al., 2022), it is well-established that participants typically exhibit an ERD phase following the cue, which is then followed by a recovery phase characterized by ERS (up to approximately 1s after the conclusion of MI). In this study, we concentrated on the MI interval, specifically 0.5 to 2.5s after the cue, a time window commonly employed in BCI classification (Ang et al., 2012).

Since this interval directly follows the cue and coincides with the MI phase, its average power change serves as a representation of ERD strength. The average ERD was calculated as shown in Equation 2:


(2) $$\begin{array}{l} {\bar{\text{E}\text{R}\text{D}}}_{i,j}^{l}\left(\text{ch}\right)=\frac{1}{N}\overset{N}{\underset{s=1}{\sum }}{\text{E}\text{R}\text{D}}_{i,j}^{l}\left(s,\text{ch}\right), \end{array}$$

Where ${\bar{\text{E}\text{R}\text{D}}}_{i,j}^{l}\left(\text{ch}\right)$ denotes the average ERD percentage computed over *N* time samples within the selected interval (0.5 to 2.5s). These computations provide an estimate of the ERD strength for each trial, forming the basis for our subsequent analysis.

### 2.3 BCI classification accuracy

In this study, we followed a commonly used BCI classification methodology for motor imagery tasks (Ramoser et al., 2000; Wang et al., 2006; Ang et al., 2008). After EEG preprocessing and 8–30 Hz bandpass filtering, EEG data from the 0.5 to 2.5-s interval following cue onset were extracted for feature computation. Spatial filtering was applied using the Common Spatial Patterns (CSP) algorithm (Ramoser et al., 2000). From the resulting spatial filters, the six most discriminative components were selected. The spatially filtered EEG signals were used to compute the variance for each trial segment, and the log-transformed variance values were then used as features. These features were input to a Linear Discriminant Analysis (LDA) classifier. Classification performance was evaluated using 10-fold cross-validation, with 90% of the data used for training and 10% for testing in each fold.

### 2.4 Statistical analysis

To investigate the relationship between pre-cue parietal alpha power and the strength of ERD at the trial level, we performed a series of statistical analyses.

First, normality was assessed using the Shapiro–Wilk test, and the assumption was violated for both ERD and alpha power distributions. Therefore, Spearman correlation coefficient was used to assess the association. For each session, Spearman correlation coefficient (*r*) was calculated to assess the association between the single-trial averaged ERD, ${\bar{\text{ERD}}}_{i,j}^{l}\left(\text{ch}\right)$ from Equation 2, and its corresponding pre-cue parietal alpha power, ${\bar{\alpha }}_{i,j}^{l}\left(\text{Pz}\right)$, across all trials. This analysis evaluated whether variations in pre-cue parietal alpha power were linked to changes in ERD strength on a trial-by-trial basis.

In addition to trial-level analyses, a group-level correlation analysis was performed by computing the median pre-cue parietal alpha power and corresponding median ERD for each participant. For participants with multiple sessions, values were averaged prior to aggregation to maintain statistical independence. A Spearman correlation was then applied to assess the association between these subject-level median values.

Next, for each session, trials were categorized into two groups based on whether their pre-cue parietal alpha power values were above or below the median value across all trials. The non-parametric Mann-Whitney U-test was then used to compare ERD strength between these high- and low-alpha power groups . This analysis aimed to identify significant differences in ERD strength associated with differing levels of pre-cue alpha activity.

Thereafter, the non-parametric Spearman correlation coefficient was used to evaluate the relationship between classification accuracy and pre-cue parietal alpha power across 77 participants. For participants with multiple sessions, alpha power and accuracy were first averaged to ensure statistical independence across observations. Classification accuracy for each participant was determined using 10-fold cross-validation, computed as the average accuracy across the 10 testing folds. Pre-cue parietal alpha power for each participant was derived from the training partitions of the 10 folds. Specifically, within each fold, the median pre-cue parietal alpha power was calculated, and these median values were averaged across all folds to obtain a single participant-level metric.

Descriptive statistics, including means and standard deviations, were computed for all key variables. Statistical significance was determined at *p* < 0.05. To control for multiple comparisons, the Benjamini–Hochberg false discovery rate (FDR) correction was applied at *q* = 0.05.

Finally, topographic maps were created to visually show how ERD patterns differ between trials with higher and lower pre-cue parietal alpha power. These visualizations were included to help explore whether different levels of pre-cue alpha power are linked to noticeable differences in ERD patterns across the scalp.

## 3 Results

### 3.1 Correlation analysis between MI -ERD and pre-cue parietal alpha power

**Figure 2:**
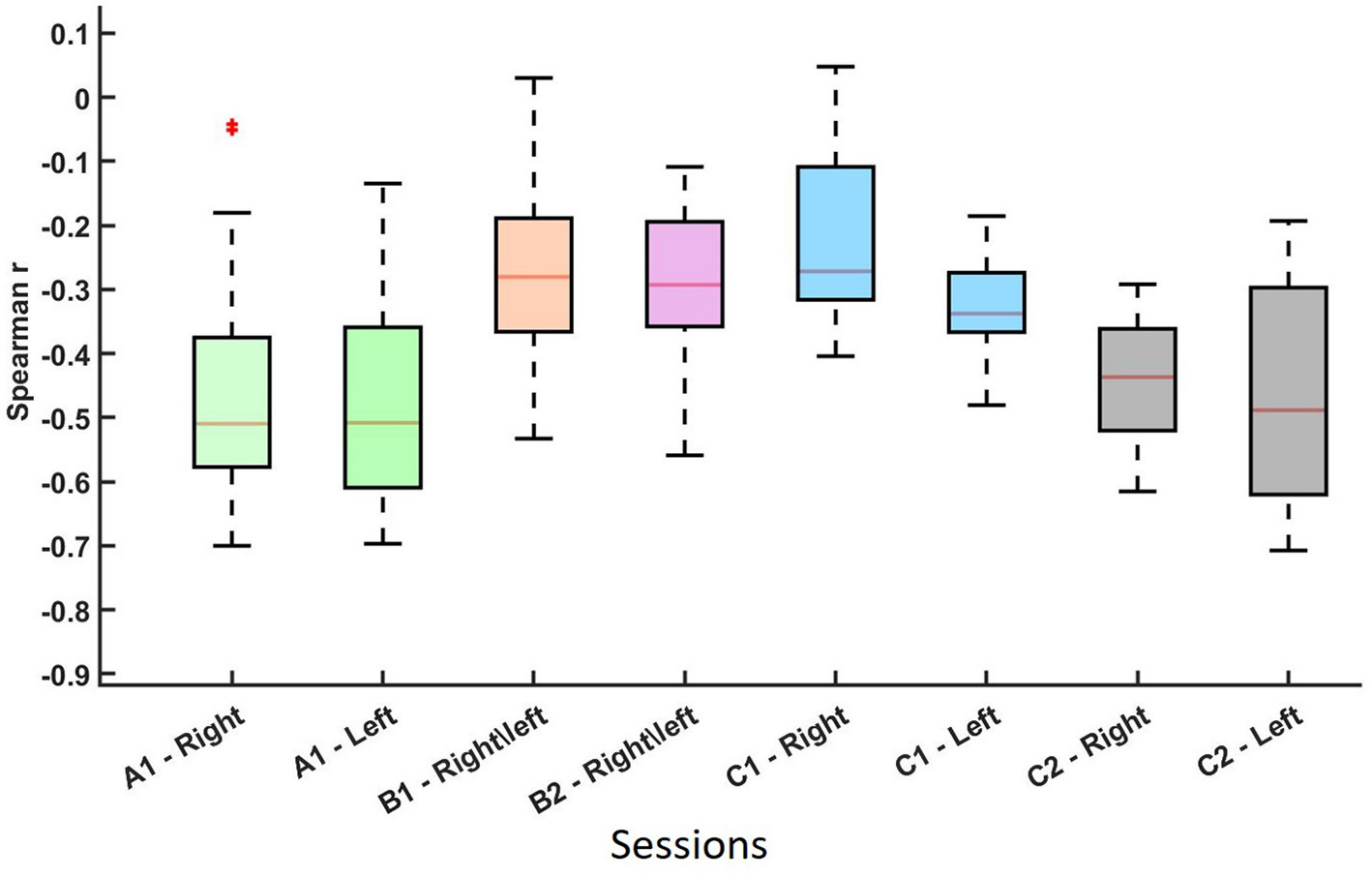
Distribution of the non-parametric Spearman correlation coefficients (r-values) between ${\bar{ERD}}_{i,j}^{l}\left(\text{at C3 for right-hand MI and C4 for left-hand MI}\right)$ and pre-cue parietal alpha power ${\bar{\alpha }}_{i,j}^{l}\left(Pz\right)$ for datasets A, B, and C across different sessions and MI conditions. Each boxplot represents a session-hand condition. These results indicate a consistent negative relationship between pre-cue alpha power and ERD strength across conditions. The asterisk (*) represents an outlier in the data distribution.

**Figure 3:**
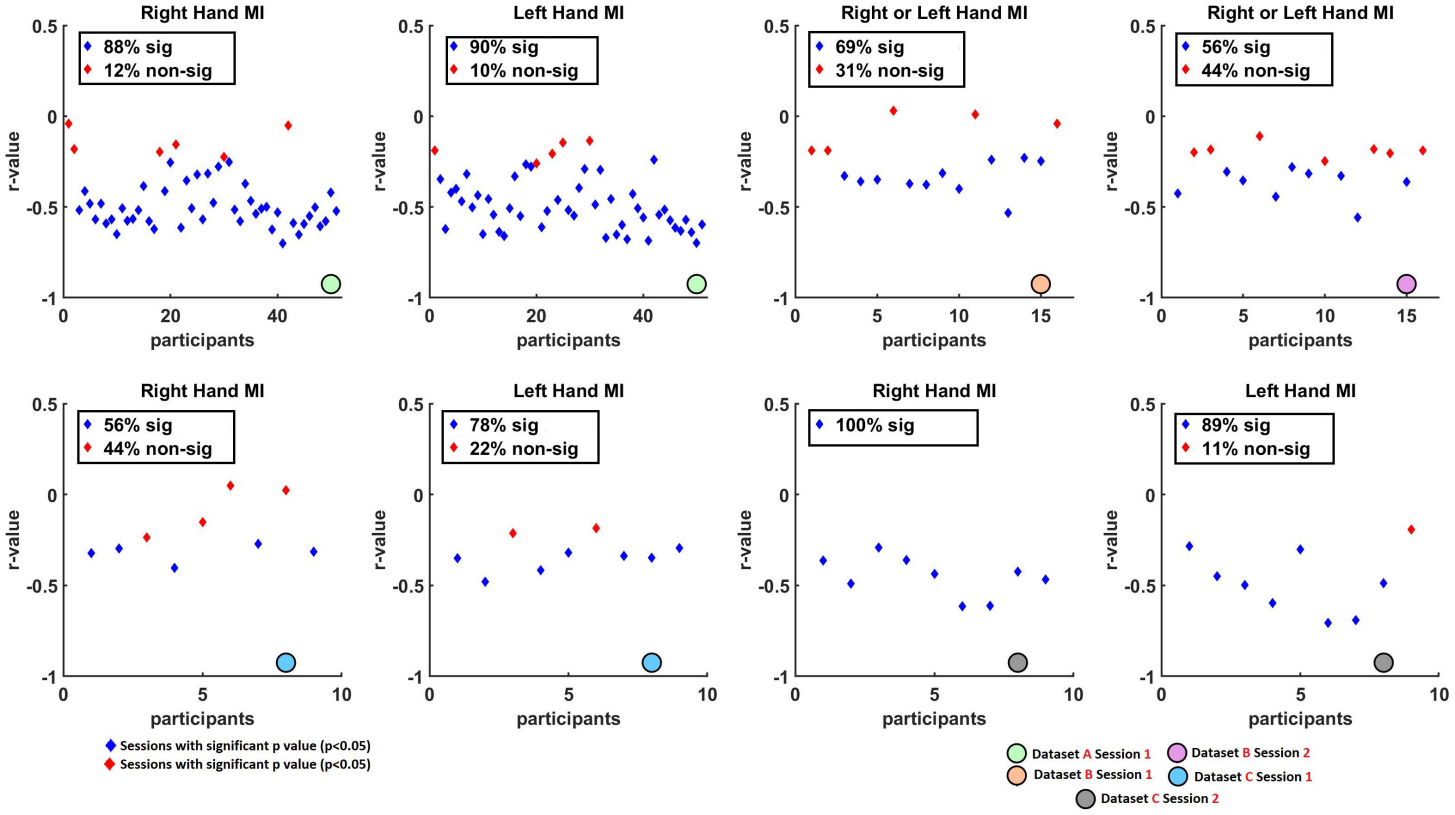
Scatter plots illustrating the non-parametric Spearman correlation coefficients (*r*-values) between single-trial ${\bar{\text{E}\text{R}\text{D}}}_{i,j}^{l}$ (at C3 for right-hand and C4 for left-hand tasks) and pre-cue parietal alpha power ${\bar{\alpha }}_{i,j}^{l}$(at Pz) across Datasets A, B, and C. Blue diamonds represent sessions with statistically significant correlations after FDR correction (*q* < 0.05), while red diamonds indicate sessions with non-significant correlations (*p*>0.05). Overall, consistent negative correlations were found across datasets, suggesting that higher pre-cue parietal alpha power is associated with stronger (i.e., more negative) ERD responses.

Spearman correlation analysis was conducted to assess the relationship between average pre-cue parietal alpha power and averaged ERD values across sessions and MI tasks. The results are visualized in Figures 2, 3, which summarize the distribution and significance of correlation coefficients across different datasets and conditions.

As shown in Figure 2 and summarized in Table 1, each session-hand condition exhibited a predominantly negative correlation between pre-cue alpha power and ERD. Values are reported in the format of median(mean ± standard deviation). In Dataset C2, the strongest and most consistent inverse associations were observed, with median *r*-values of −0.437 (−0.451 ± 0.110) for right-hand MI and −0.488 (−0.468 ± 0.181) for left-hand MI. Dataset A showed similarly strong effects, with medians of −0.510 (−0.466 ± 0.151) and −0.508 (−0.477 ± 0.122) for right- and left-hand MI, respectively. Greater variability was observed in Dataset B, where the median *r*-values were −0.281 (−0.258 ± 0.092) in Session B1 and −0.293 (−0.293 ± 0.184) in Session B2. Dataset C1 showed moderate effects with median values of −0.272 (−0.214 ± 0.157) and −0.338 (−0.327 ± 0.091) for right- and left-hand MI.

**Table 1:** Median, mean, and standard deviation of spearman correlation coefficients between single-trial pre-cue parietal alpha power and the corresponding ERD, averaged across all participants and sessions for each motor imagery task in Datasets A, B, and C.

**Dataset**	**Task condition**	**Median *r***	***r* (Mean ± SD)**
A	Right-hand MI	−0.510	−0.466 ± 0.151
	Left-hand MI	−0.508	−0.477 ± 0.122
B1	Right/left MI (session 1)	−0.281	−0.258 ± 0.092
B2	Right/left MI (session 2)	−0.293	−0.293 ± 0.184
C1	Right-hand MI	−0.272	−0.214 ± 0.157
	Left-hand MI	−0.338	−0.327 ± 0.091
C2	Right-hand MI	−0.437	−0.451 ± 0.110
	Left-hand MI	−0.488	−0.468 ± 0.181

Fisher's Z-transformation was applied prior to averaging.

Figure 3 presents scatter plots, highlighting the proportion of sessions that yielded statistically significant correlations (*p* < 0.05, blue diamonds) vs. non-significant results (*p*>0.05, red diamonds). High rates of significance were observed in most datasets. Specifically, in Dataset A, 88% of right-hand and 90% of left-hand MI sessions showed significant correlations. In Dataset B, Session 1 and Session 2 showed 69% and 56% of sessions with significant correlations, respectively. In Dataset C, 56% of right-hand and 78% of left-hand sessions were significant in Session 1, while Session 2 showed 100% significance for right-hand MI and 89% for left-hand MI.

**Figure 4:**
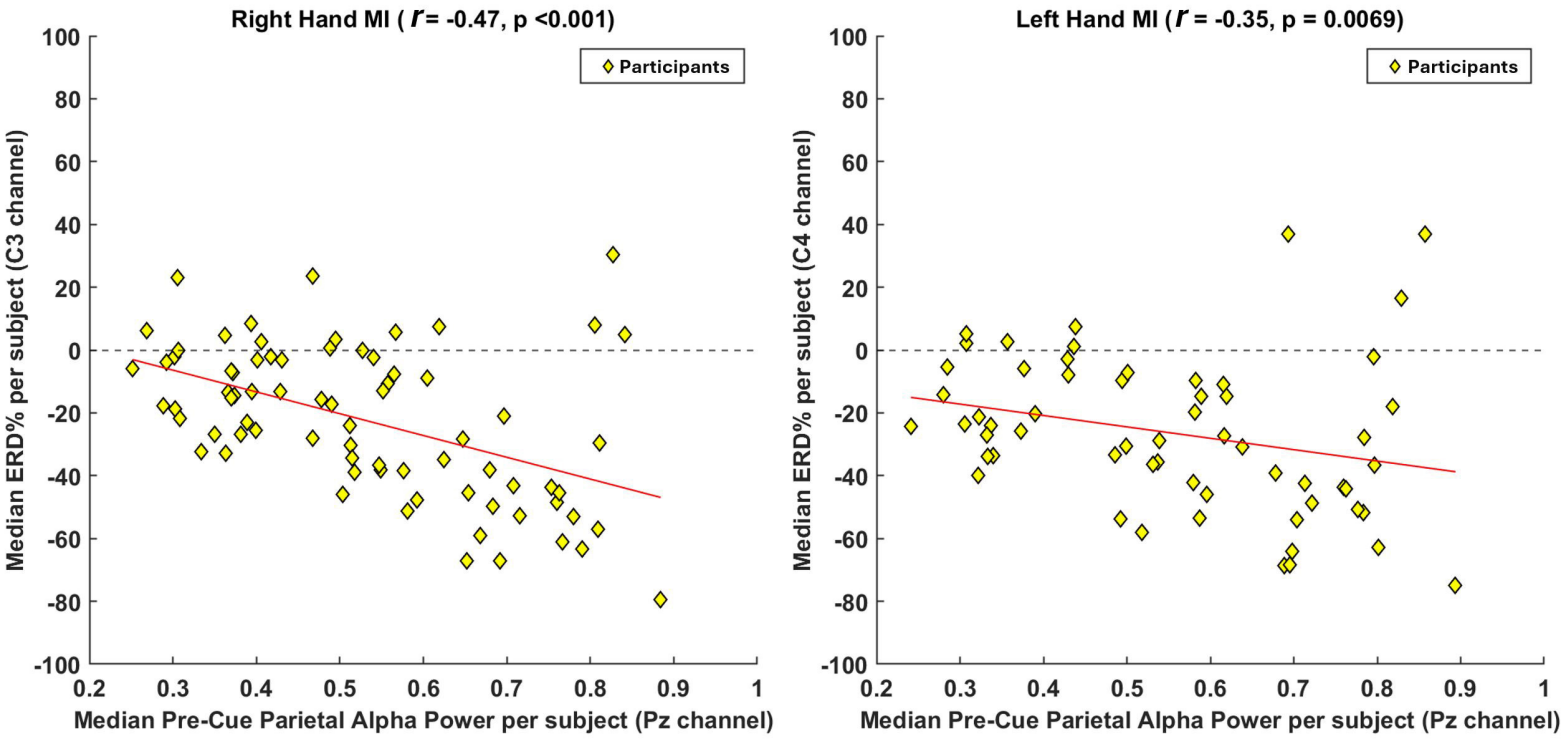
Group-level correlation analysis between pre-cue parietal alpha power and ERD strength across participants. To assess the overall participant-level trend, we computed the median pre-cue parietal alpha power (at Pz) and the median ERD values (at C3 for right-hand MI and C4 for left-hand MI) across all participants. For participants with multiple sessions, values were averaged prior to inclusion to ensure statistical independence. A non-parametric Spearman's rank correlation was applied to evaluate the association between these aggregated medians. Each yellow diamond represents one participant, and the red line denotes the fitted correlation trend. The results reveal a negative correlation, indicating that participants with higher pre-cue parietal alpha power tend to exhibit stronger ERD responses during MI.

To further examine group-level effects, Figure 4 presents participant-level median correlations. Here, the median pre-cue parietal alpha power (at Pz) and the corresponding median ERD (at C3 for right-hand MI, and C4 for left-hand MI) were computed per participant. A non-parametric Spearman's rank correlation revealed a statistically significant negative relationship for both tasks. For right-hand MI, the correlation was *r* = −0.47, *p* < 0.001, and for left-hand MI, *r* = −0.35, *p* = 0.0069. These findings confirm that, at the participant level, higher median pre-cue alpha power is associated with stronger ERD responses, further reinforcing the trial-level results and suggesting that preparatory brain states exert consistent influence across individuals.

### 3.2 Comparing ERD strengths between trials with high and low pre-cue parietal alpha power

To further explore the relationship between pre-cue parietal alpha power and ERD strength, we divided the trials for each session into two subgroups: those with higher and lower pre-cue parietal alpha power, based on the median alpha power value across the session. Given the observed negative correlation between ERD and alpha power, we hypothesized that trials with higher pre-cue parietal alpha power would exhibit a stronger ERD response. To test this hypothesis, we used the non-parametric Mann-Whitney U-test to compare ERD responses between the two subgroups, as the assumption of normality was violated, as described in Section 2.1.

Tables 2–4 present the outcomes of the Mann-Whitney U-tests. The results show that the majority of participants demonstrated a significant difference (*p* < 0.05) in ERD values between the two groups. Specifically, in Dataset A, 95% of the sessions showed significant differences in right-hand MI tasks, and 94% in left-hand MI tasks. For Dataset B, 75% of the sessions showed significant differences in Session 1, and 68% in Session 2. Similarly, in Dataset C, 67% of the sessions showed significant differences in right-hand MI tasks and 88% in left-hand MI tasks in Session 1, while 77% of the sessions showed significant differences in both right-hand and left-hand MI tasks in Session 2.

**Table 2:** Comparison of the ERD strength between two subgroups of trials for each participant and MI task in Dataset A, using the non-parametric Mann–Whitney U-test.

**Part**	**RH**	**LH**	**Part**	**RH**	**LH**
*A*_1_	0.313	0.008	*A*_27_	<0.001	<0.001
*A*_2_	<0.001	<0.001	*A*_28_	0.064	0.002
*A*_3_	<0.001	<0.001	*A*_29_	0.642	0.258
*A*_4_	<0.001	<0.001	*A*_30_	0.002	<0.001
*A*_5_	<0.001	<0.001	*A*_31_	<0.001	<0.001
*A*_6_	<0.001	<0.001	*A*_32_	<0.001	<0.001
*A*_7_	<0.001	<0.001	*A*_33_	<0.001	<0.001
*A*_8_	<0.001	<0.001	*A*_34_	0.003	<0.001
*A*_9_	<0.001	<0.001	*A*_35_	<0.001	<0.001
*A*_10_	<0.001	<0.001	*A*_36_	<0.001	<0.001
*A*_11_	<0.001	<0.001	*A*_37_	<0.001	<0.001
*A*_12_	<0.001	<0.001	*A*_38_	<0.001	<0.001
*A*_13_	<0.001	<0.001	*A*_39_	<0.001	<0.001
*A*_14_	<0.001	<0.001	*A*_40_	<0.001	<0.001
*A*_15_	<0.001	0.021	*A*_41_	<0.001	<0.001
*A*_16_	<0.001	<0.001	*A*_42_	<0.001	<0.001
*A*_17_	0.034	0.046	*A*_43_	0.702	0.012
*A*_18_	<0.001	0.003	*A*_44_	<0.001	<0.001
*A*_19_	0.055	0.088	*A*_45_	<0.001	<0.001
*A*_20_	0.028	<0.001	*A*_46_	<0.001	<0.001
*A*_21_	<0.001	<0.001	*A*_47_	<0.001	<0.001
*A*_22_	0.013	0.011	*A*_48_	<0.001	<0.001
*A*_23_	<0.001	<0.001	*A*_49_	<0.001	<0.001
*A*_24_	0.001	0.054	*A*_50_	<0.001	<0.001
*A*_25_	<0.001	<0.001	*A*_51_	<0.001	<0.001
*A*_26_	<0.001	<0.001	*A*_52_	<0.001	<0.001

Trials were divided into two subgroups based on their pre-cue parietal alpha power: the upper alpha power group includes trials with pre-cue parietal alpha power above the median, while the lower alpha power group includes trials below the median. The calculation of the median for the pre-cue parietal alpha powers was session and task specific.

**Table 3:** Comparison of the ERD strength between two subgroups of trials for each participant, and session in Dataset B, using the non-parametric Mann–Whitney U-test.

**Part**	***P*-value session 1**	***P*-value session 2**
*B*_1_	0.059	<0.001
*B*_2_	0.005	0.228
*B*_3_	0.145	0.005
*B*_4_	0.002	0.045
*B*_5_	0.004	0.145
*B*_6_	0.418	0.520
*B*_7_	<0.001	0.002
*B*_8_	0.016	0.005
*B*_9_	0.059	0.004
*B*_10_	0.005	0.005
*B*_11_	0.145	0.418
*B*_12_	0.002	0.012
*B*_13_	0.004	<0.001
*B*_14_	0.418	0.020
*B*_15_	<0.001	0.016
*B*_16_	0.016	0.566

Please note that participants in Dataset B performed only one MI task. Trials were divided into two subgroups based on their pre-cue parietal alpha power: the upper alpha power group includes trials with pre-cue parietal alpha power above the median, while the lower alpha power group includes trials below the median. The calculation of the median for the pre-cue parietal alpha powers was session and task specific.

**Table 4:** Comparison of the ERD strength between two subgroups of trials for each participant, session and task in Dataset C, using the non-parametric Mann–Whitney U-test.

**Part**	**Session 1**		**Session 2**	
	**RH**	**LH**	**RH**	**LH**
*C*_1_	0.025	0.002	0.009	<0.001
*C*_2_	0.126	0.060	0.006	0.001
*C*_3_	0.115	0.002	0.561	0.210
*C*_4_	0.019	0.030	0.265	0.037
*C*_5_	0.083	0.020	0.002	0.121
*C*_6_	<0.001	<0.001	0.005	<0.001
*C*_7_	0.009	<0.001	<0.001	0.004
*C*_8_	<0.001	<0.001	<0.001	<0.001
*C*_9_	0.002	<0.001	<0.001	0.051

Trials were divided into two subgroups based on their pre-cue parietal alpha power: the upper alpha power group includes trials with pre-cue parietal alpha power above the median, while the lower alpha power group includes trials below the median. The calculation of the median for the pre-cue parietal alpha powers was session and task specific.

**Figure 5:**
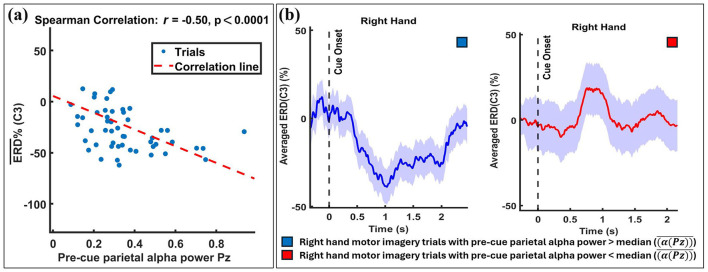
Exemplary results from a single participant (B_13, 1_) illustrating the relationship between pre-cue parietal alpha power and ERD. Trials were divided into two subgroups based on the median pre-cue parietal alpha power: the *upper* subgroup (trials with pre-cue alpha power above the median) and the *lower* subgroup (trials with pre-cue alpha power below the median). **(a)** A significant negative correlation (*r* = −0.50, *p* < 0.001) is shown between single-trial pre-cue alpha power (at Pz) and ERD during the right-hand MI task (at C3), with individual trials represented by blue circles and the linear regression line in red. **(b)** Temporal evolution of ERD at C3 for the two subgroups, with shaded regions indicating the standard deviation across trials. The results demonstrate that higher pre-cue alpha power is associated with stronger ERD.

The statistical results are reflected in the visual data. Figure 5 provides an example from participant B_13, 1_. The figure begins by showing the strength of the Spearman correlation between single-trial ERD values and their corresponding pre-cue parietal alpha powers, as depicted in the scatter plot in Figure 5a. A significant negative correlation (*r* = −0.50, *p* < 0.001) between pre-cue parietal alpha power and ERD in the C3 channel for right-hand MI tasks is observed. Figure 5b displays the trials split by the median pre-cue parietal alpha power into upper and lower groups, showing the temporal evolution of the ERD response over the MI period. The subplot indicates that trials in the upper group (higher pre-cue alpha power) resulted in stronger ERD (more negative values), while the lower group showed no considerable ERD during the MI task.

**Figure 6:**
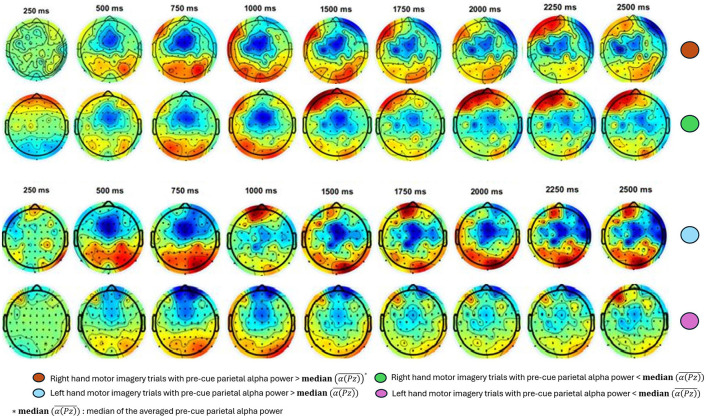
Exemplary results for topographic maps of ERD over time for right- and left-hand MI trials for Participant A_1, 3_. Each column represents a different time point, ranging from 250 ms to 2500 ms post-cue. The trials are divided into two subgroups based on the median of the averaged pre-cue parietal alpha power. ERD is notably stronger (indicated by more pronounced blue) in the upper-half group for both hands, suggesting greater neural engagement and desynchronization compared to the lower-half group.

Similarly, Figure 6 presents another example of the ERD response between the two median split groups for the Participant A_3_. The topographic maps of ERD for both right- and left-hand MI trials cover time points from 250 ms to 2500 ms after the cue. These maps reveal that trials with higher pre-cue alpha power show more pronounced ERD prominent in the contralateral motor cortex, indicating greater neural engagement and desynchronization compared to trials with lower pre-cue alpha power.

### 3.3 Localization of alpha, ERD association across EEG channels

To investigate whether the observed relationship between pre-cue parietal alpha power and motor imagery-related ERD was spatially localized or diffusely distributed, we performed a channel-wise Spearman correlation analysis. For each participant, we computed the correlation between alpha power (8-12 Hz) at each EEG channel and the corresponding ERD in the contralateral sensorimotor cortex (C3 for right-hand MI, C4 for left-hand MI). For participants who completed two sessions, correlations were averaged within participant to preserve statistical independence.

**Figure 7:**
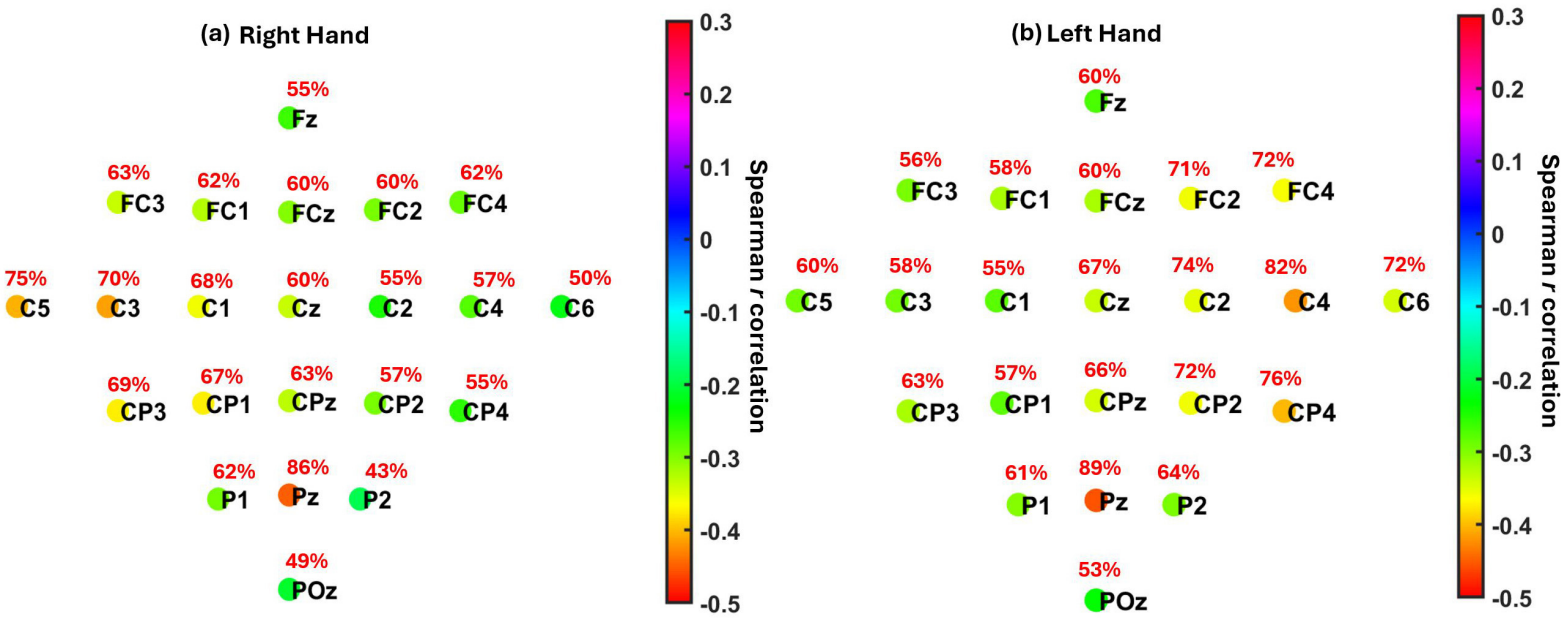
Localization of the association between pre-cue parietal alpha power and MI-related ERD across EEG channels. **(a)** Right hand MI. **(b)** Left hand MI. Each electrode is color-coded by the median Spearman correlation coefficient (*r*) across participants between pre-cue alpha power (8–13 Hz) cross all channels and ERD strength at the contralateral motor cortex (C3 for right hand, C4 for left hand). Red text indicates the percentage of participants showing statistically significant correlations at each electrode after FDR correction (*q* = 0.05). The results show maximum negative correlations were observed at Pz (*r* = −0.45 for right hand, *r* = −0.46 for left hand) and at contralateral motor electrodes C3 (*r* = −0.42) and C4 (*r* = −0.42), respectively.

Group-level patterns were then visualized using scalp maps (Figure 7), which depict (1) the median Spearman's *r* across participants at each channel, and (2) the percentage of participants with statistically significant correlations at each location, following FDR correction (*q* = 0.05). For both right- and left-hand MI tasks, the strongest correlations were consistently observed at parietal site Pz (median *r* = −0.45 and −0.46, respectively), where a large proportion of participants also showed significant effects (86% and 89%). The next strongest correlations were found at C3 and C4, the contralateral sensorimotor sites, with slightly weaker median *r* values and lower proportions of significance. The corresponding summary of participant-level significance percentages is reported in Table 5.

**Table 5:** Summary of median correlation coefficients and percentage of participants with significant correlations between pre-cue alpha power and ERD across EEG channels.

**Task**	**Peak channel**	**Median *r***	**% Sig. parti**	**Next-strongest channel**	**Median *r***	**% Sig. parti**
Right hand MI	Pz	-0.45	86%	C3	-0.42	70%
Left hand MI	Pz	-0.46	89%	C4	-0.42	82%

These findings support the presence of a distinct parietal alpha source driving the observed relationship, rather than a diffuse or volume-conducted effect from the sensorimotor cortex. The parietal focus is consistent with the role of posterior alpha in anticipatory attention and preparatory states. Meanwhile, the secondary effects observed over C3/C4 likely reflect local contributions from baseline μ rhythms in M1/S1, in line with their expected involvement in contralateral motor planning and control.

### 3.4 Association between pre-cue parietal alpha power and BCI classification accuracy

In this subsection, we examined whether the median pre-cue parietal alpha power extracted from training data was associated with BCI classification accuracy on testing data. As outlined in Section 2.4, the median pre-cue parietal alpha powers from the 10 training folds of each session were averaged and compared with the corresponding average 10-fold BCI classification accuracy.

**Figure 8:**
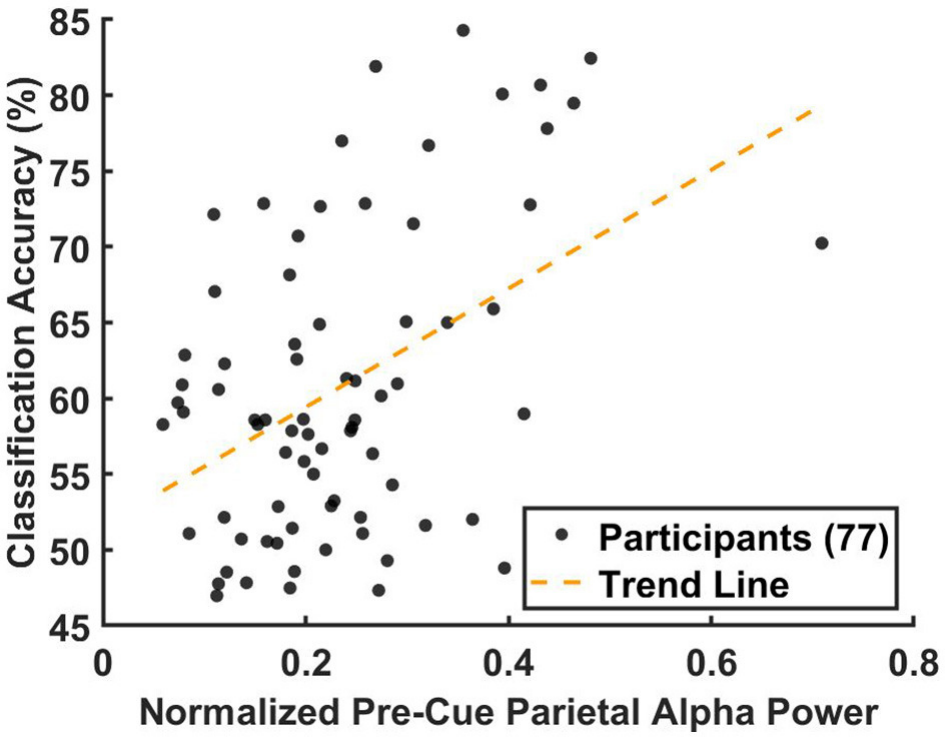
Correlation between the median pre-cue parietal alpha power from the training data and the corresponding BCI classification accuracy from the testing data, analyzed across 77 participants. For participants with multiple sessions, results were averaged prior to the correlation analysis to ensure statistical independence. Pre-cue alpha power was normalized by dividing each trial's alpha power by the total power in the 8–40 Hz band to account for inter-dataset variability. For each participant, classification accuracy was computed using 10-fold cross-validation and averaged across folds. Each participant is represented as a black circle, with the yellow dashed line indicating the linear regression fit. The non-parametric Spearman correlation analysis revealed a significant association (*r* = 0.344, *p* < 0.0027).

Figure 8 shows the results of the non-parametric Spearman correlation analysis, evaluating the relationship between the median parietal alpha power, ${\bar{\alpha }}_{i,j}^{l}\left(Pz\right)$, and the 10-fold cross-validation BCI classification accuracy across 77 participants. Since the analysis was conducted at the group level rather than the participant level, pre-cue alpha power for each trial was normalized by dividing it by the total power. This normalization ensured that data from all three datasets were comparable, accounting for differences in baseline alpha power levels caused by factors such as variations in amplifiers and recording equipment.

The results, presented in Figure 8, revealed a significant positive association between the normalized average pre-cue parietal alpha power and classification accuracy (Spearman's *r* = 0.344, *p* < 0.0027). A corresponding linear regression indicated that pre-cue alpha power explained approximately 12% of the variance in classification accuracy (*R*^2^≈0.12).

## 4 Discussion and conclusion

In summary, this study investigated how variations in pre-cue parietal alpha power influence the magnitude of ERD during MI tasks, as well as how they relate to the overall MI-BCI classification accuracy.

By analyzing 102 sessions from 77 participants across three datasets, we consistently observed a negative correlation, indicating that higher pre-cue parietal alpha power is linked to stronger ERD. This suggests that a more relaxed preparatory brain state, reflected by elevated pre-cue parietal alpha activity, may facilitate the generation of more robust MI-related desynchronization. Previous studies have linked high parietal alpha power to the suppression of distracting information and improved allocation of attentional resources (Klimesch, 2012; Sokoliuk et al., 2019; Pfurtscheller and Da Silva, 1999; Jensen and Mazaheri, 2010), potentially supporting readiness for task execution (Foxe and Snyder, 2011). In our study, this preparatory alpha state was consistently associated with stronger ERD, implying that the attentional or inhibitory functions of alpha power may support more pronounced sensorimotor engagement during motor imagery.

Building on this, we also found a statistically significant—though moderate—positive association between pre-cue parietal alpha power and MI-BCI classification accuracy. At first glance, this may appear to contrast with findings from Bamdadian et al. (2014), who reported that a combination of lower parietal alpha, lower central beta, and higher frontal theta was associated with better classification performance. Their interpretation suggested that reduced alpha reflects heightened attentional engagement, which in turn supports improved MI-BCI control. However, it is important to note that their analysis used a composite predictor, and did not assess the independent contribution of parietal alpha power in isolation from beta and theta activity.

Even though our correlation with classification accuracy was moderate and explained around 12% of the variance, most previously proposed neurophysiological predictors, including those by Bamdadian et al. (2014); Ahn and Jun (2015), have failed to replicate reliably, as highlighted by Jeunet et al. (2015). These studies relied on classification accuracy as a performance metric, which is complex and affected by many external factors (Lotte and Jeunet, 2017), as discussed earlier. Our study interprets that shifts in attention could influence the generation of ERD and may not be accurately captured by a single averaged classification. Attention needs to be a measurable and dynamic marker that reflects moment-to-moment brain state changes. This helps explain when strong ERD is produced and when it is not, arguably offering a stronger and more direct indicator of user performance with fewer confounding parameters than classification accuracy.

In addition to neurophysiological factors, previous studies have indeed identified psychological traits—such as motivation, confidence, anxiety, and cognitive control—as significant predictors of MI-BCI performance (Jeunet et al., 2015; Nijboer et al., 2010). Importantly, several of these psychological factors have also been linked to parietal alpha power in the broader neuroscience literature. For example, increased parietal alpha is associated with internalized attention, reduced anxiety, and greater cognitive readiness (Klimesch, 1999; Angelakis et al., 2004; Sauseng et al., 200; Foxe and Snyder, 2011). These psychological states are conducive to better MI-BCI control and align with the preparatory role we propose for pre-cue alpha. Notably, non-neurophysiological predictors have been shown to explain substantial variance in BCI performance,up to 30% for concentration and motor skills (Hammer et al., 2012), and approximately 35% for locus of control when dealing with technology (Burde and Blankertz, 2006). Jeunet et al. (2015) reported that a combination of cognitive and personality traits could account for over 80% of performance variability. While we did not assess psychological traits directly in our dataset, our finding that higher pre-cue parietal alpha predicts stronger motor-related desynchronization is consistent with this literature and suggests that pre-cue alpha may serve as an objective, neurophysiological proxy for a broader set of psychological readiness factors.

Building on this distinction, the observed association between pre-cue parietal alpha power and MI-BCI performance also underscores the potential for more personalized MI-BCI training programs. Traditional approaches often overlook individual neurophysiological differences, leading to inconsistent outcomes. Incorporating pre-cue alpha power into training protocols allows for customized strategies, such as adjusting cue timing, modifying feedback, or scaling task difficulty to match each user's mental state. While human observers typically rely on indirect behavioral cues (e.g., reaction times, posture, or facial expressions) to gauge user engagement, alpha-based metrics provide a direct and continuous neural readout of cognitive readiness. In principle, such real-time neural indicators could inform more consistent and objective adjustments during training–such as when to pause a trial, adapt feedback, or recalibrate task demands based on the user's fluctuating attentional state. For example, users with lower pre-cue parietal alpha power may benefit from extended preparation time, relaxation activities before the session, or more frequent feedback, whereas those with higher alpha power may thrive with increased task difficulty and greater cognitive challenges.

While the observed correlations between pre-cue parietal alpha power, ERD strength, and classification accuracy are robust and consistent, further work is needed to better understand the nature of these relationships. As alpha power was not experimentally manipulated in this study, the findings cannot yet confirm whether increased pre-cue alpha actively facilitates stronger ERD responses or simply reflects a favorable underlying cognitive state. Future studies using interventional approaches–such as neurofeedback to enhance alpha activity or closed-loop BCI systems that adjust task timing based on real-time alpha fluctuations–could help establish directionality and provide stronger evidence for the role of pre-cue parietal alpha as a modifiable factor in MI-BCI performance. In addition, while pre-cue parietal alpha power was moderately associated with classification accuracy at the group level, in some cases participants with multiple sessions (e.g. dataset B), the session with the highest alpha power also yielded the highest classification accuracy. In several cases (e.g., B8, B9, B13), the difference in alpha power across sessions was small, whereas accuracy varied more substantially. Future work could further investigate this dissociation using larger datasets to better characterize the relationship between stable neurophysiological traits and session-specific performance fluctuations in MI-BCI tasks.

## Data Availability

The original contributions presented in the study are included in the article/supplementary material, further inquiries can be directed to the corresponding author.
